# A Pitfall of Wheezing – A Large Mediastinal Mass Presenting as Persistent Wheezing: A Case Report

**DOI:** 10.7759/cureus.21340

**Published:** 2022-01-17

**Authors:** Kenji Iwai, Kenichi Tetsuhara, Satoshi Tsuji, Mitsuru Kubota

**Affiliations:** 1 Division of Emergency and Transport Services, National Center for Child Health and Development, Tokyo, JPN; 2 Department of Paediatrics, Kitahara Neurosurgical Institute, Tokyo, JPN; 3 Department of Critical Care Medicine, Fukuoka Children’s Hospital, Fukuoka, JPN; 4 Department of General Pediatrics and Interdisciplinary Medicine, National Center for Child Health and Development, Tokyo, JPN

**Keywords:** position, ecmo, mediastinal mass, wheezing, anchoring bias

## Abstract

Anchoring bias is the tendency to pursue only the most salient feature, which can lead to closed-minded thinking in the early stage of the diagnostic process. Wheezes are one of the most frequent chief complaints and highly likely to become an anchoring bias. We described a patient initially receiving a diagnosis of asthma after presenting with persistent wheezes; however, there was no improvement upon treatment for asthma and eventually, an anterior mediastinal mass was found. The patient’s respiratory condition suddenly deteriorated when he was placed in a prone position and eventually extracorporeal membrane oxygenation (ECMO) was introduced. We must recognize the danger of anchoring bias with any symptoms. A wheezing patient with an atypical clinical course should undergo further investigations, given the possibility of other etiologies such as a mediastinal tumor. In addition, we have to pay close attention to the patient’s position when a mediastinal tumor is suspected.

## Introduction

Anchoring bias is the tendency to pursue only the most salient feature, which can lead to closed-minded thinking in the early stage of the diagnostic process [1]. Since wheezes are one of the most frequent chief complaints in the pediatric emergency department [2] and usually most of them are caused by respiratory infection and asthma [3], it is highly likely to become an anchoring bias. We described a patient initially receiving a diagnosis of asthma after presenting with persistent wheezes caused by a mediastinal tumor and finally found to be suffering from respiratory distress due to the gravitational compression of the trachea and vessels.

## Case presentation

**Figure 1:**
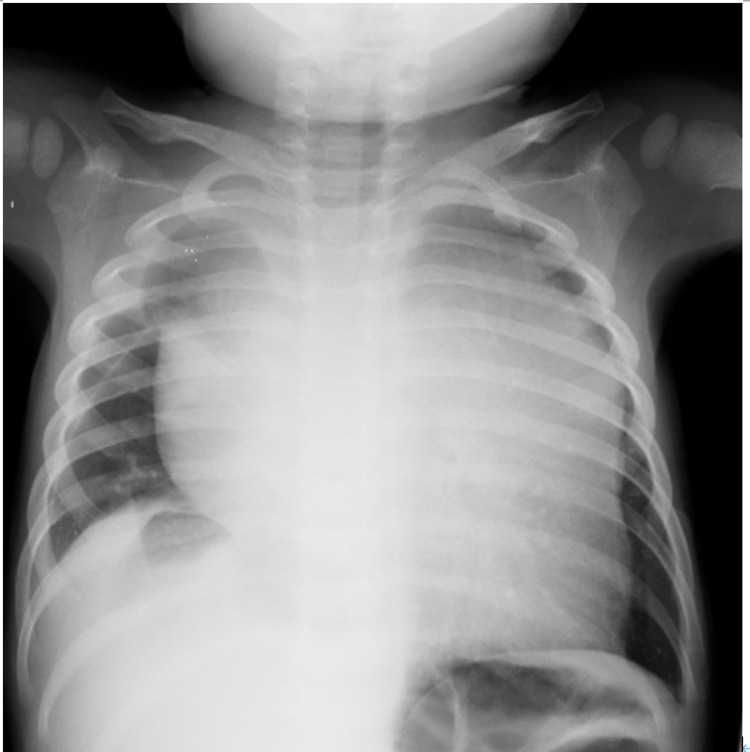
Chest X-ray Chest X-ray shows expanded mediastinal shadow. This is the first chest X-ray taken.

The patient is a two-year-old boy with no past medical history. Two weeks prior to admission, he developed wheezing and cough and was examined by his family physician. His condition was diagnosed as asthma, although his symptoms did not improve with inhaled β-agonist. His symptoms gradually exacerbated three days prior to admission. On the day of admission, he was referred to a nearby hospital because he showed respiratory distress with chest retraction and wheezing. On arrival, his SpO_2_ level was 92% on room air, and he had chest retraction and wheezing. Oxygen administration was immediately initiated. However, his symptoms deteriorated in the supine position when physicians tried to obtain intravenous access. A chest X-ray in the standing position revealed an enlarged mediastinal shadow (Figure 1).

**Figure 2:**
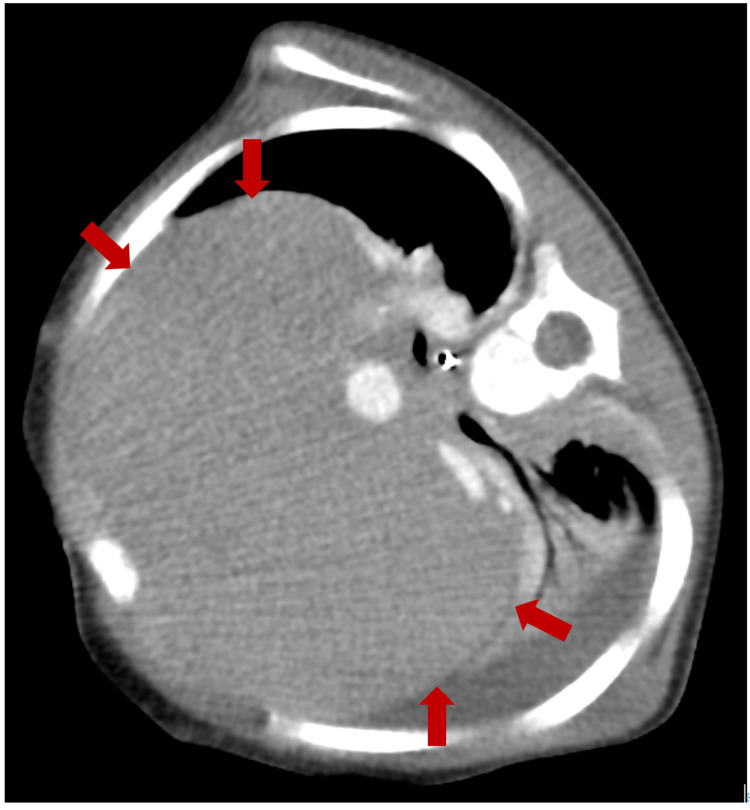
Contrast-enhanced CT of chest Contrast-enhanced CT of the chest reveals an anterior mediastinal tumor measuring 114×74×97 cm.

Laboratory data were as follows: white blood cell count 274,000/µL, hemoglobin 9.0 g/dL, platelet count 3,600/µL. The results of venous blood gas analysis were as follows: pH 7.08, PCO_2_ 60.7 torr, lactate 7.2 mmol/L, base excess -14.0 mEq/L. Heart failure was suspected due to the enlarged mediastinal shadow and he was transferred to a regional tertiary care hospital. Vital signs on arrival were as follows: heart rate of 160 beats per minute and SpO_2_ of 96% on room air. Physical examination revealed he had modest respiratory distress and both inspiratory and expiratory wheezing. The patient was placed in a lateral position to improve his respiratory condition. The chest contrast-enhanced computed tomography showed a large anterior mediastinal mass (Figure 2).

**Figure 3:**
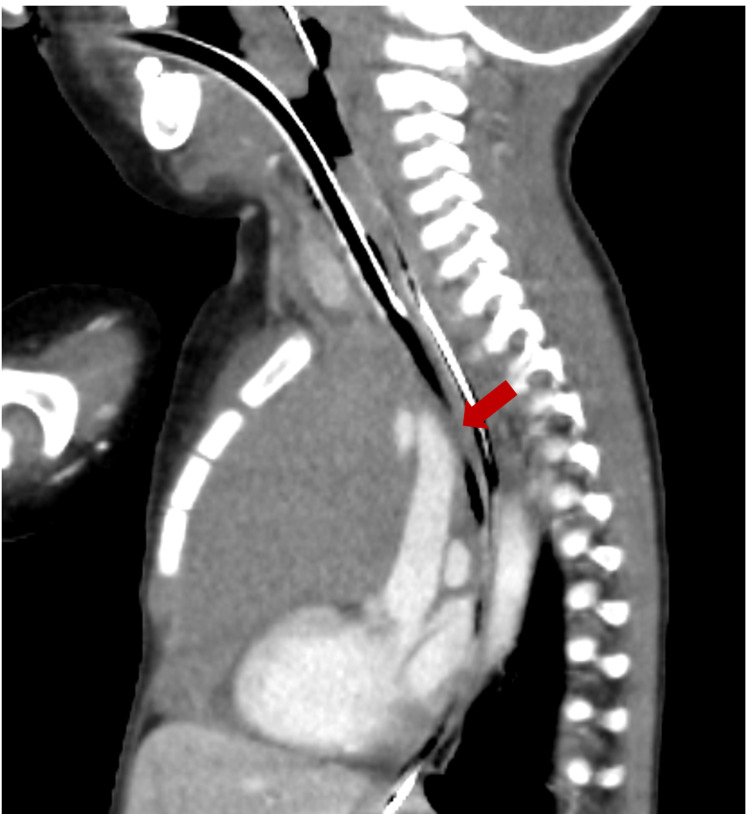
Contrast-enhanced CT The trachea distal to the intubation tube collapsed due to compression by the tumor.

**Figure 4:**
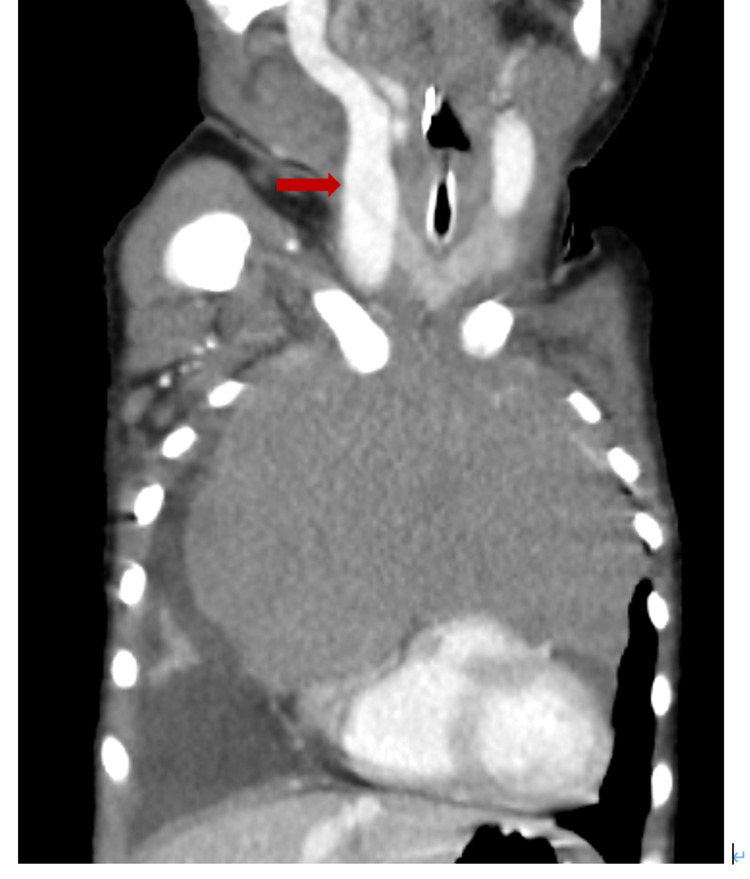
Contrast-enhanced CT Internal jugular veins are distended due to the compression of the superior vena cava.

It also showed narrowing of the trachea, bronchi deviated posteriorly, and distended bilateral internal jugular veins resulting from compression of the superior vena cava by the mass (Figures 3, 4).

Lymphoma, thymoma and leukemia were considered as differential diagnoses for anterior mediastinal tumor. Among these differential diagnoses, leukemia was strongly suspected based on the result of blood test. Therefore, bone marrow aspiration was performed to confirm the diagnosis. During bone marrow aspiration, SpO_2_ level dropped to 88% and the heart rate also gradually decreased to 58 beats per minute in the prone position. His heart rate was restored after cardiopulmonary resuscitation for three minutes, and endotracheal intubation and mechanical ventilation were introduced. Even under mechanical ventilation, pulmonary function was impaired due to the compression of the trachea and bronchi by the tumor. He was transferred to a tertiary care center for the management of respiratory failure by extracorporeal membrane oxygenation (ECMO). As the tumor was found to have originated from T-cell acute lymphoblastic leukemia according to the result of bone marrow aspiration, chemotherapy was initiated. On the fifth day of admission, ECMO treatment was discontinued and high flow nasal cannula (HFNC) was introduced. On the ninth day of admission, HFNC was terminated and the patient was shifted to oxygen administration only. The mediastinal tumor had shrunk over time. He continues to be on chemotherapy without any sequelae.

## Discussion

We described a patient initially receiving a diagnosis of asthma after presenting with persistent wheeze; however, there was no improvement upon treatment for asthma and eventually an anterior mediastinal mass was found originating from T-cell acute lymphoblastic leukemia. The patient’s respiratory condition suddenly deteriorated when he was placed in a prone position and eventually ECMO was introduced due to severe cardiopulmonary complication, despite invasive mechanical ventilations. In this case, we learned the following two important clinical points: the danger of anchoring bias when assessing a wheezing patient and paying special attention to the patient’s position when we see a patient with a suspected mediastinal mass.

Regarding the first clinical point, physicians must recognize the danger of anchoring bias, which is one of the most frequent diagnostic biases [4]. Anchoring bias is the tendency to pursue only the most salient feature, which can lead to closed-minded thinking in the early stage of the diagnostic process [1]. This could also lead to misdiagnosis and eventually result in a life-threatening condition. In fact, wheezing is one of the most frequent chief complaints in the pediatric emergency department [2] and usually most of them are caused by respiratory infection and asthma [3]. It is therefore highly likely to become an anchoring bias, such as in this case. The patient was treated as asthma for more than one week despite being unresponsive to the treatments such as inhaled β-agonist and his condition was finally diagnosed as a large mediastinal mass compressing the trachea and bronchi. Aside from asthma and respiratory infection, wheezing is also caused by heart failure [5], foreign body aspiration, vascular ring, and rarely triggered by a mediastinal mass [6]. Chest X-ray should be considered when a patient with wheezing is unresponsive to the treatment for asthma or the symptoms are persistent. Chest X-ray was reported to have a high sensitivity of 97% for detecting mediastinal mass [7]. Although it is not recommended to routinely obtain a chest X-ray for a patient with the first episode of wheezing when the diagnosis of bronchiolitis or asthma is clear [8], it should be done when the clinical course is complex or the diagnosis is unclear. Chest X-ray could be helpful to detect not only a mediastinal tumor, but also to obtain findings of heart failure or atelectasis. It is crucial to identify a patient with wheezing caused by a mediastinal mass as early as possible because this can lead to fatal cardiopulmonary complications [9,10]. Learning about and recognizing cognitive biases is definitely the very first step in order to avoid a diagnostic error.

Regarding the second clinical point, physicians must be careful of the patient’s position when we see a patient with suspected mediastinal mass because serious cardiopulmonary complications may occur as a result of gravitational extrinsic compression of the airway and major vessels [11]. There are case reports about patients suffering serious cardiopulmonary complications due to a large mediastinal mass [9,10,12]. To date, the management of patients with a mediastinal mass for general anesthesia has been well established [13]. Even before general anesthesia, careful preoperative risk assessment including determining the most comfortable position for the patient is recommended, especially for infants [14]. Although the risk evaluation and management of a patient with suspected mediastinal mass in the emergency department setting has not been well studied, it is obvious that physicians have to pay special attention to the patient’s position.

## Conclusions

In conclusion, we must recognize the danger of anchoring bias when assessing wheezing. A wheezing patient with an atypical clinical course and a poor response to asthma treatments should undergo further investigations, such as chest X-ray, given the possibility of other etiologies such as a mediastinal tumor aside from asthma and respiratory infection. In addition, we have to pay close attention to the patient’s position when a mediastinal tumor is suspected because the respiratory condition can dramatically deteriorate due to the gravitational compression of the trachea and vessels.

## References

[1] Croskerry P (2003). The importance of cognitive errors in diagnosis and strategies to minimize them. Acad Med.

[2] Scarfone RJ, Luberti AA, Mistry RD (2004). Children referred to an emergency department by an after-hours call center: complaint-specific analysis. Pediatr Emerg Care.

[3] Ducharme FM, Tse SM, Chauhan B (2014). Diagnosis, management, and prognosis of preschool wheeze. Lancet.

[4] Saposnik G, Redelmeier D, Ruff CC, Tobler PN (2016). Cognitive biases associated with medical decisions: a systematic review. BMC Med Inform Decis Mak.

[5] Tetsuhara K, Tsuji S, Nakano K, Kubota M (2017). Heart failure in dilated cardiomyopathy mimicking asthma triggered by pneumonia. BMJ Case Rep.

[6] Kliegman RM, Stanton B, St. Geme J, Schor N (2016). Nelson Textbook of Pediatrics, 20th Edition. Nelson Textbook of Pediatrics 20th edition. Amsterdam, the Netherland: Elsevier.

[7] Harris GJ, Harman PK, Trinkle JK, Grover FL (1987). Standard biplane roentgenography is highly sensitive in documenting mediastinal masses. Ann Thorac Surg.

[8] Kathy N, Richard G (2016). Fleisher & Ludwig’s Textbook of Pediatric Emergency Medicine, 7th Edition. Fleisher & Ludwig’s Textbook of Pediatric Emergency Medicine 7th editon. Philadelphia, PA: Wolters Kluwer.

[9] Piastra M, Ruggiero A, Caresta E, Chiaretti A, Pulitano S, Polidori G, Riccardi R (2005). Life-threatening presentation of mediastinal neoplasms: report on 7 consecutive pediatric patients. Am J Emerg Med.

[10] Lam JC, Chui CH, Jacobsen AS, Tan AM, Joseph VT (2004). When is a mediastinal mass critical in a child? An analysis of 29 patients. Pediatr Surg Int.

[11] Pearson JK, Tan GM (2015). Pediatric anterior mediastinal mass: a review article. Semin Cardiothorac Vasc Anesth.

[12] Suominen PK, Kanerva JA, Saliba KJ, Taivainen TR (2010). Unrecognized mediastinal tumor causing sudden tracheal obstruction and out-of-hospital cardiac arrest. J Emerg Med.

[13] Hack HA, Wright NB, Wynn RF (2008). The anaesthetic management of children with anterior mediastinal masses. Anaesthesia.

[14] Tütüncü AÇ, Kendigelen P, Kaya G (2017). Anaesthetic management of a child with a massive mediastinal mass. Turk J Anaesthesiol Reanim.

